# Hot Electrons Control of Quantum Dot Emission Using Plasmonic Supercells

**DOI:** 10.1021/acs.jpcc.6c03809

**Published:** 2026-07-13

**Authors:** Seyed M. Sadeghi, Rithvik Gutha, Christina Sharp, Ryan W. Goul, Judy Wu

**Affiliations:** † Department of Physics and Astronomy, 14843 University of Alabama in Huntsville, Huntsville, Alabama 35899, United States; ‡ Department of Physics and Astronomy, The University of Kansas, Lawrence, Kansas 66045, United States

## Abstract

In hybrid systems composed of metallic nanoantennas and semiconductor quantum dots (QDs), exciton–plasmon coupling plays a central role in controlling the emission intensity and dynamics of the QDs. Here, we investigate how plasmon decay into hot electrons modulates this coupling through electronic modification of the QD environment. The plasmonic platform consists of closely packed periodic arrays of elongated Au nanoantennas that support plasmonic supercells. Owing to their periodicity, these supercells, formed via near-field coupling along the long axes or through hybrid plasmonic–photonic edge coupling, support plasmonic hot-spots and surface lattice resonances (SLRs), supporting enhanced hot-electron generation. The arrays are coated with a thin Si interlayer followed by an ultrathin InP/ZnS QD film, providing both dielectric coupling and electronic pathways for charge transfer. We show that hot electrons injected across the Au/Si interface can charge the environment of QDs, resulting in a polarization-dependent blue shift of the emission of QDs, accompanied by enhancement of their emission lifetime. These results highlight the key roles of SLRs and plasmonic hot spots in enabling hot-electron-mediated control of QD emission.

## Graphical abstract

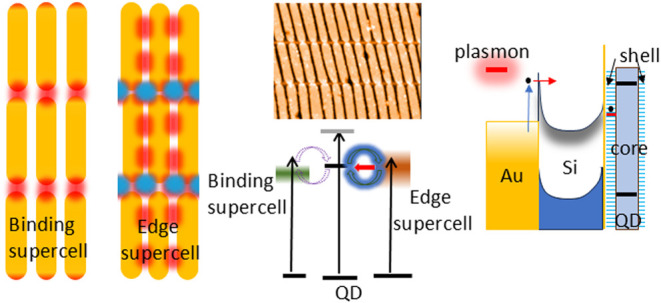

## Introduction

Periodic arrays of metallic nanostructures support rich optical features, including strongly localized plasmonic hot-spots and collective resonances known as surface lattice resonances (SLRs).^1,2^ Plasmonic hot-spots originate from intense near-field confinement within individual nanostructures, whereas SLRs arise from long-range diffractive coupling among periodically arranged scatterers.^2–4^ Architectures that enable the coexistence and mutual interaction of these effects are of particular interest, as they provide simultaneous access to localized field enhancement and collective coherence. Different techniques have been utilized to study such architectures. These include arrays of dimers,^5^ complex metallic structure units,^6^ and periodic arrays of regions containing nanoislands and colloidal metallic nanoparticles.^7^ They also include periodic arrays of plasmonic supercells–unit cells composed of relatively long chains of near and/or far field coupled nanoantennas.^8^

Plasmonic supercells form when elongated, flat nanoantennas (F-nanos) are placed in close proximity along their long axis (x), giving rise to polarization-dependent coupling pathways. Under x-polarized illumination, near-field interactions link adjacent antennas in an end-to-end configuration, forming bonding supercells (B-SCs), as illustrated in Figure 1a. In contrast, y-polarized excitation redirects coupling toward the antenna corner edges, leading to the formation of edge supercells (E-SCs, Figure 1b,1c). Periodic repetition of these E-SCs enables diffractive coupling across the array, resulting in ”edge” surface lattice resonances (e-SLRs). Such SLRs combine localized plasmonic fields with plasmonic-photonic edge coupling (Figure 1c).

**Figure 1:**
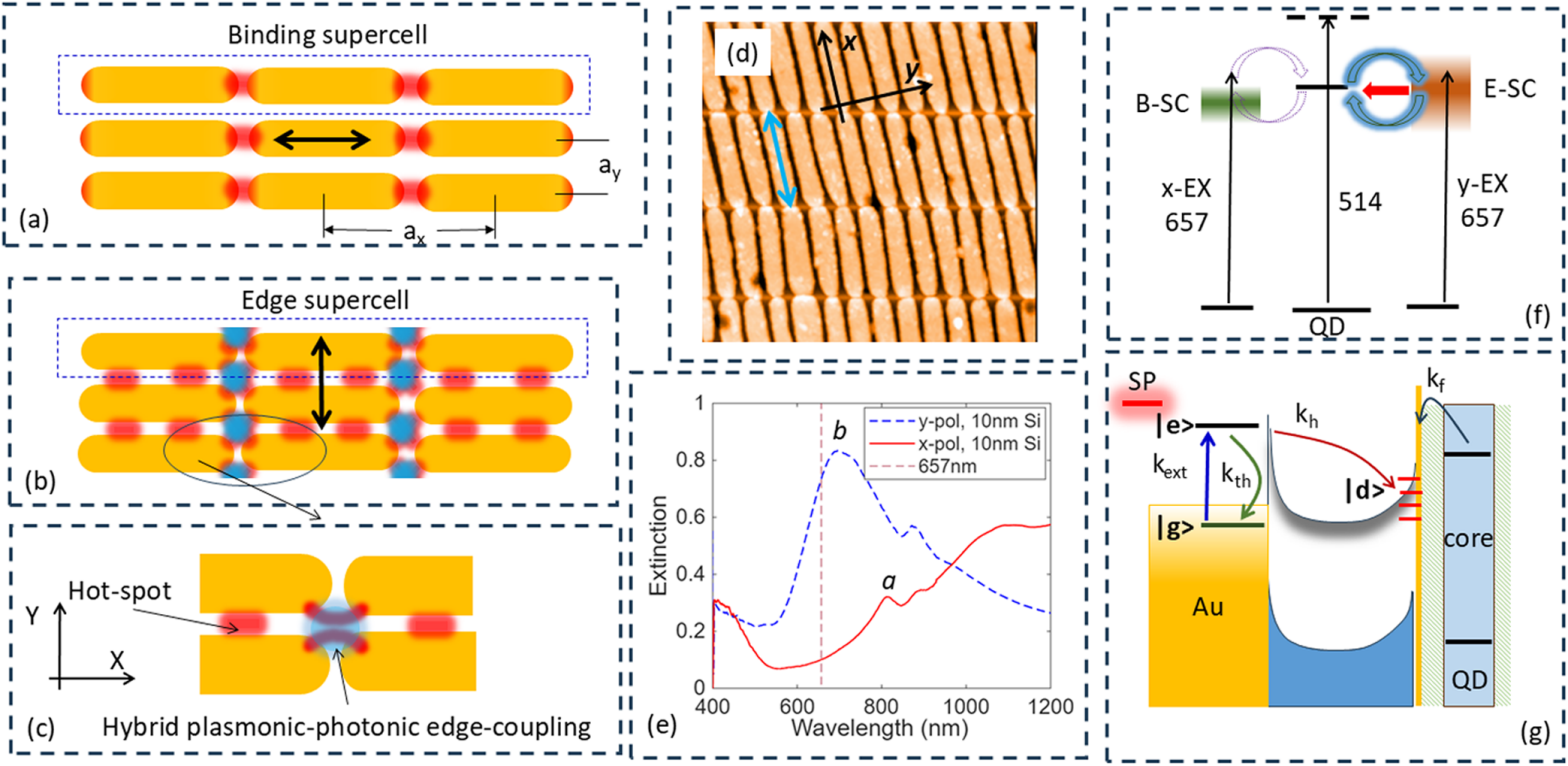
Schematics of the binding supercell (B-SC) (a), edge supercell (E-SC) (b), and a detailed view of the hybrid photonic–plasmonic edge coupling responsible for E-SC formation (c). (d) AFM image of the double-side d array; the two-sided arrow scale corresponds to 1 μm. (e) Extinction spectra of the array with the Si interlayer for incident light polarized along the *x*-axis (solid line) and *y*-axis (dashed line). (f) Excitation configuration, and (g) schematic illustration of hot-electron generation and subsequent QD charging. The two-sided arrows in (a) and (b) refer to polarization of incident light.

SLRs are characterized by the coherent excitation of surface plasmon resonances (SPRs) within the constituent nanoantennas. Such coherently driven SPRs can decay nonradiatively, generating energetic charge carriers, including hot electrons.^9,10^ These electrons may overcome interfacial potential barriers and inject into adjacent semiconductors or molecular layers,^11^ enabling a variety of plasmon-assisted processes such as photocatalysis, photodetection, solar energy conversion, and modulation of quantum emitters.^12^ Collective plasmonic modes, and in particular SLRs, are therefore expected to play an important role in enhancing hot-electron generation by coherently concentrating plasmonic excitation over extended length scales.^13–15^

Here, we investigate hot-electron–mediated interactions in hybrid systems consisting of InP/ZnS QD films interfaced with arrays of F-nanos designed to support SCs and e-SLRs. The nanoantenna arrays are engineered such that the e-SLR spectrally overlaps with the QD emission band, while a thin sputtered Si interlayer provides both dielectric coupling and an electronic pathway between the Au nanoantennas and the QDs. By probing QD emission under off-resonant and near-resonant excitation, we examine how polarization-selective excitation of B-SC and E-SC influences exciton–plasmon interaction and hot-electron generation. We show that hot electrons injected across the Au/Si interface can influence QD emission ny electrostatically charging their environment. This in turn leads to a polarization-dependent blue shift of the QDs’ emission and enhancement of their emission lifetime. These results highlight the key roles of SLRs and plasmonic hot-spots in enabling directional exciton–plasmon coupling and hot-electron–mediated control of QD emission, establishing a framework for exploring the impact of hot-electrons in plasmonic systems and optoelectronic devices.

## Methodology and Characterization

Electron-beam lithography was used to fabricate arrays of Au F-nanos on a glass substrate. The nanoantenna dimensions were 950 nm along the *x*-axis, 220 nm along the *y*-axis, and approximately 40 nm in height. The lattice constants along these axes, denoted as *a*_*x*_ and *a*_*y*_, were 1000 and 300 nm, respectively. The arrays were patterned in four regions, each with an area of 200 μm × 200 μm, and were subsequently coated with a 10 nm-thick Si layer using sputtering. Figure 1d shows an AFM image of the sample prior to Si deposition. Subsequently, InP/ZnS QDs (aquired from NN Laboratories Inc.) were spin-coated onto the Si layer after dilution in toluene at a volume ratio of 1:10 (QD:toluene). The InP/ZnS QDs are estimated to have core sizes in the range of approximately 3.5–5.8 nm, with an average size of 4.5 nm.

The measured extinction spectra of the arrays are shown in Figure 1e. When the incident light was polarized along the *x*-axis, the spectrum exhibited a broad resonance with several minor peaks (red solid line) with the first peak at 808 nm representing a B-SC (peak *a*). In contrast, for y-polarized illumination, a distinct and intense peak (peak b) appeared near 700 nm (blue solid line), corresponding to e-SLR.

To elucidate the formation of SCs and e-SLRs, we performed full-wave electromagnetic simulations using Lumerical (2025). Figure 2a shows the top-view (*x*–*y* plane) geometry of the simulated structure, consisting of Au nanoantennas with dimensions and periodicity matching those of the experimentally fabricated arrays on a glass substrate. The superstrate is considered to have with effective refractive index of 1.1, to include the impact of the Si layer. Figure 2b presents the simulated extinction spectra for incident light polarized along the *x*-axis (solid line) and *y*-axis (dashed line). Overall, the simulated response reproduces the experimental trends observed in Figure 1e. The discrepancies are attributed primarily to the absence of surface roughness and fabrication-induced disorder in the simulations.

**Figure 2:**
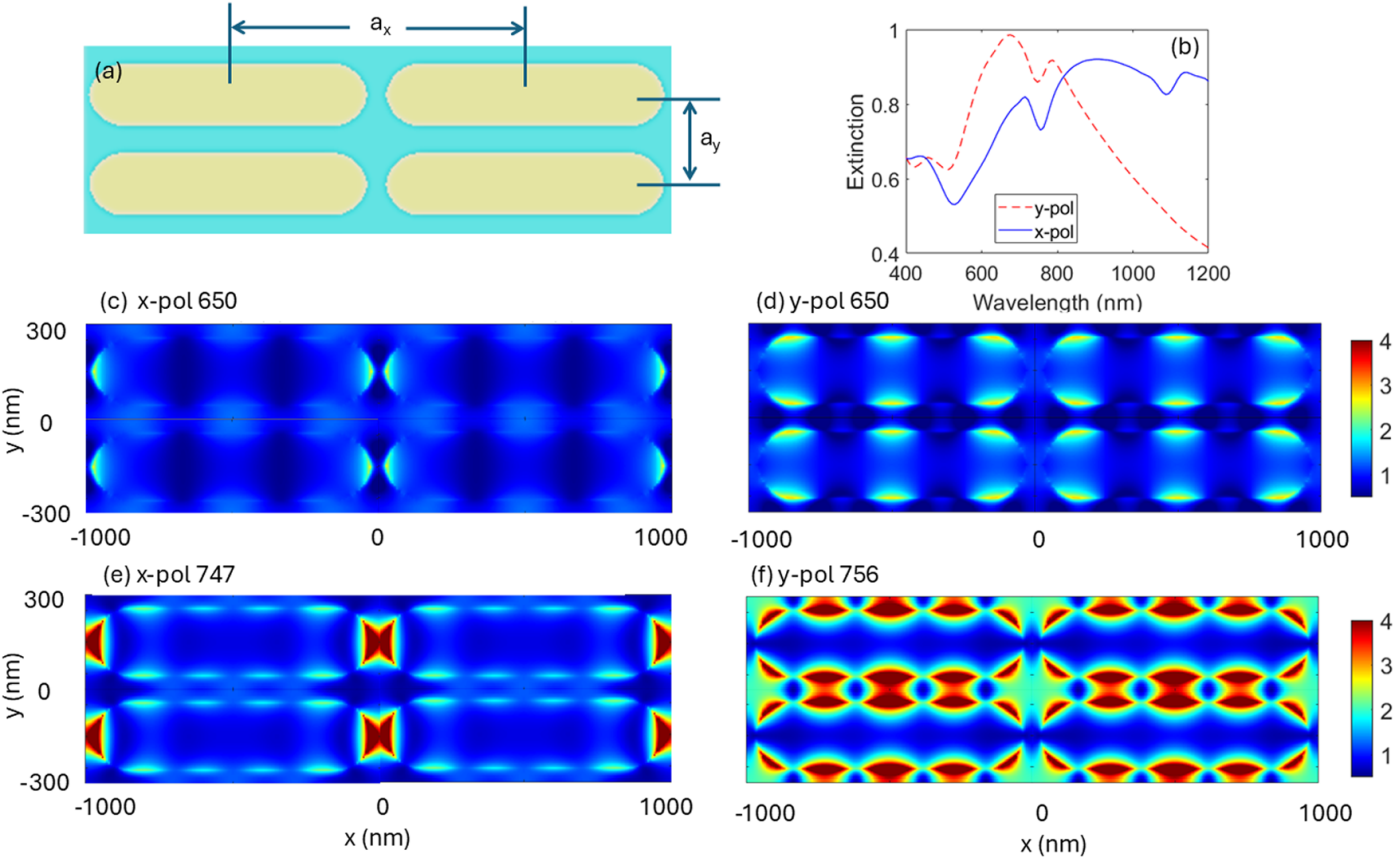
(a) Top-view geometry of the simulated structures. (b) Simulated extinction spectra for incident light polarized along the *x*-axis (solid line) and *y*-axis (dashed line). (c) and (d): Field enhancement distributions in the *x*–*y* plane for *x*- and *y*-polarized excitation at around 650 nm. (e) and (f): Corresponding field enhancement distributions for *x*- and *y*-polarized excitation at 747 and 758 nm, respectively. The *x*–*y* plane passed through the top of F-nanos at a distance of 10 nm from the from the top of the nanoantennas. The color-coded bars to the right represent the range of field enhancement factor. In (a) *a*_*x*_ = 1000 nm and *a*_*y*_ = 300 nm refer to the lattice constants.

Figure 2c,2d show the spatial distribution of the field enhancement factor in the *x*–*y* plane located at 10 nm above the top of the F-nanos, i.e., 50 nm from glass substrate, at a wavelength of about 650 nm. The field enhancement is defined as the ratio of the squared electric field intensity in the presence of the nanoantennas to that in their absence. At this wavelength, the enhancement is mainly due to localized edge and tip modes, which are concentrated at the antenna ends and side edges, depending on the polarization of the incident light.^16–18^ In the case of x-pol the end edges have higher field concentration while in the case of y-pol the side edges support more field enhancement.

At longer wavelengths, specifically 747 nm (Figure 2e) and 756 nm (Figure 2f), the near-field distributions show collective coupling between neighboring nanoantennas, indicating the formation of SCs. For x-polorized excitation at 747 nm, the field distribution is consistent with B-SC formation driven by end-to-end near-field coupling along the *x*-axis (Figure 1a). In contrast, excitation at 756 nm with y-pol leads to strong edge-localized fields that couple adjacent nanoantennas along both the *x*- and *y*-axes, consistent with the formation of E-SCs (Figure 1b,1c). This behavior suggests a second-order orthogonal Rayleigh coupling process, in which excitation with y-pol light supports diffractive coupling along the *y*-axis. Notably, Figure 2f shows the simultaneous presence of hot-spots and coherent edge-to-edge coupling mediated by the optical field, which is a signature of e-SLR. Figure 1c schematically illustrates this process in more detail, where edge-to-edge near fields are hybridized with photonic lattice modes.

Figure 3a illustrates the experimental configuration, in which the excitation polarization was aligned either along the *x*-axis (x-EX) or the *y*-axis (y-EX). The QD emission was analyzed using a polarization analyzer with its transmission axis oriented at an angle (θ_*p*_) relative to the *x*-axis, corresponding to the longitudinal direction of the F-nanos. Steady-state photoluminescence measurements were performed using a continuous-wave 514 nm laser, spectrally detuned from the SC resonances to minimize direct plasmonic excitation. Time-resolved photoluminescence measurements were carried out using a 657 nm pulsed laser with a pulse width of 30 ps and a time-correlated single-photon counting (TCSPC) system (PicoQuant TimeHarp 260 PICO). The emitted photons were spectrally selected by a monochromator and detected using a Becker & Hickl PMC-150 single-photon photomultiplier tube (s-PMT), operated with a Becker & Hickl DCC-100 controller.

**Figure 3:**
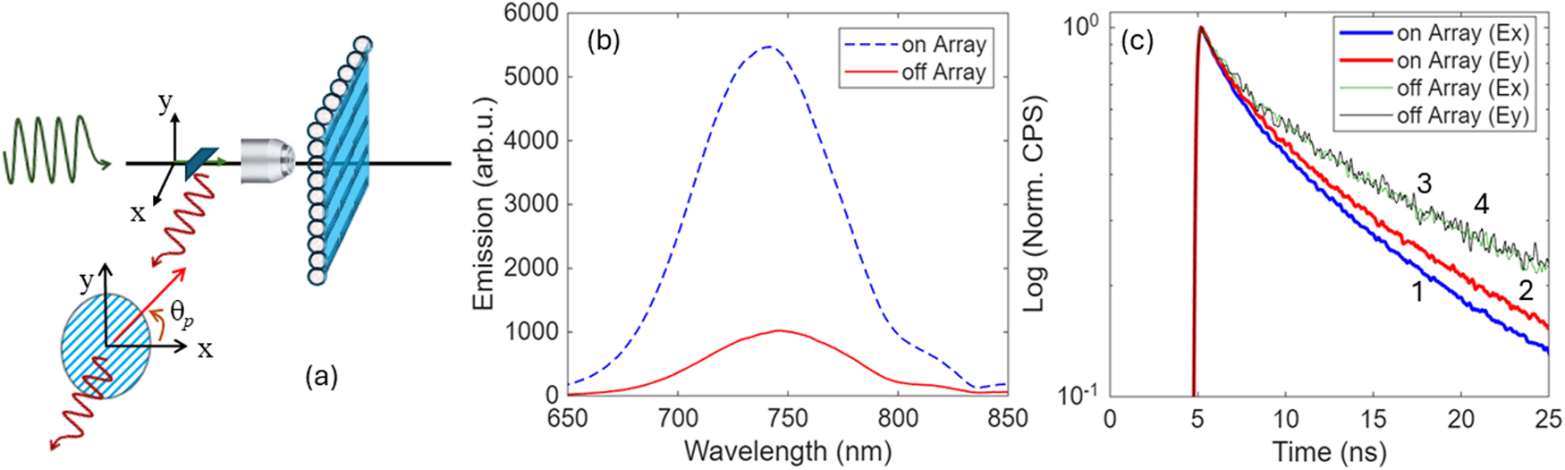
(a) Optical setup used for QD emission measurements. (b) Emission spectra of QDs on a glass/Si substrate (solid line) and on the F-nano array (dashed line) under continuous-wave excitation at 514 nm. (c) QD decay dynamics on the nanoantenna array under 657 nm excitation with x-polarized (x-EX, line 1) and y-polarized (y-EX, line 2) light. Lines 3 and 4 in (c) show the corresponding decay dynamics for QDs on the glass/Si substrate.

The temporal response of the TCSPC system is governed by the combined contributions of the laser pulse width, the detector transit time spread, and the timing electronics. Based on the manufacturer’s specifications of these components and assuming statistically independent Gaussian timing contributions, the overall instrument response function (IRF) is estimated to be approximately 140 ps (fwhm). Since the shortest fluorescence lifetime measured in this work is 1.66 ns, which is more than an order of magnitude longer than the estimated IRF, the reported decay dynamics are well within the temporal resolution of the measurement system.

## QD Emission in the Presence of B-SC and E-SC

As schematically illustrated in Figure 1f, E-SCs and B-SCs can be selectively addressed using y-EX and x-EX, respectively. For excitation at 514 nm, however, the incident laser is spectrally far from the surface plasmon resonances of both the individual nanoantennas and the SCs. As a result, no significant polarization-dependent plasmonic enhancement during the excitation process is expected. In fact under these conditions, the SCs are excited indirectly through excitons formed after energy relaxation of photoexcited electrons and holes to the band edges of the QDs. In contrast, the 657 nm laser used for x-EX and y-EX can directly excite the B-SC and E-SC modes, respectively, while simultaneously exciting the QDs (Figure 1f). This direct plasmonic excitation, as discussed below, strongly influences the generation and impact of hot electrons.

Under the off-resonant excitation condition by 514 nm laser, the emission spectra of QDs deposited on a glass/Si substrate and on the nanoantenna array are compared in Figure 3b. The QDs on the F-nano array (dashed line) exhibit a markedly higher emission intensity than those on the substrate (solid line). The emission peak appears near 740 nm, suggesting that the observed enhancement can be associated primarily with coupling to the E-SC and e-SLR during the emission process, with nearly no plasmonic impact during optical excitation. Note that for Figure 3b we removed the polarization analyzer shown in Figure 8a, measuring unpolarized emission.

Lines 1 and 2 in Figure 3c show the emission decay of QDs on the F-nano array at 740 nm, excitated by the 657 nm pulsed laser polarized along the *x* axis (x-Ex) and *y* axis (y-Ex), respectively. Lines 3 and 4 correspond to the same measurements performed on the glass/Si substrate. The decay dynamics of curves 3 and 4 are nearly identical, indicating negligible impact of laser polarization on QDs decay. In contrast, curves 1 and 2 exhibit clear differences in the decay behavior on the F-nano array. Considering the polarization-selective excitation of B-SC and E-SC, these results indicate that y-Ex, which preferentially addresses E-SC (Figure 1f), leads to a slower decay compared to that caused by x-Ex.

To further investigate the coupling between the array and the QDs, a polarization analyzer was introduced into the collection path, as illustrated in Figure 3a, with its transmission axis aligned either along the *x*-axis (the long axis of the F-nanos) or the *y*-axis (the short axis). In Figure 3a, θ_*p*_ = 0° corresponds to emission polarized along the *x*-axis, while θ_*p*_ = 90° corresponds to emission polarized along the *y*-axis. As in the measurements of Figure 3b, the QDs were excited using a 514 nm laser.

The spectra shown in Figure 4a reveal that the x-polarized emission (x-QD, solid line) experiences a stronger enhancement, whereas the y-polarized emission (y-QD, dashed line) exhibits not only enhancement but also a distinct blue shift. In contrast, for QDs deposited on the glass/Si substrate, the emission spectra were independent of the analyzer orientation, confirming the unpolarized nature of their emission (dash-dotted line). These observations are summarized in Figure 4b, which presents the emission enhancement factors obtained by normalizing the x-QD and y-QD emission intensities to those measured from regions without arrays (glass/Si). The results show that the enhancement factor of y-QD exceeds that of x-QD at wavelengths below 725 nm, consistent with the blue-shifted emission spectrum of y-QD.

**Figure 4:**
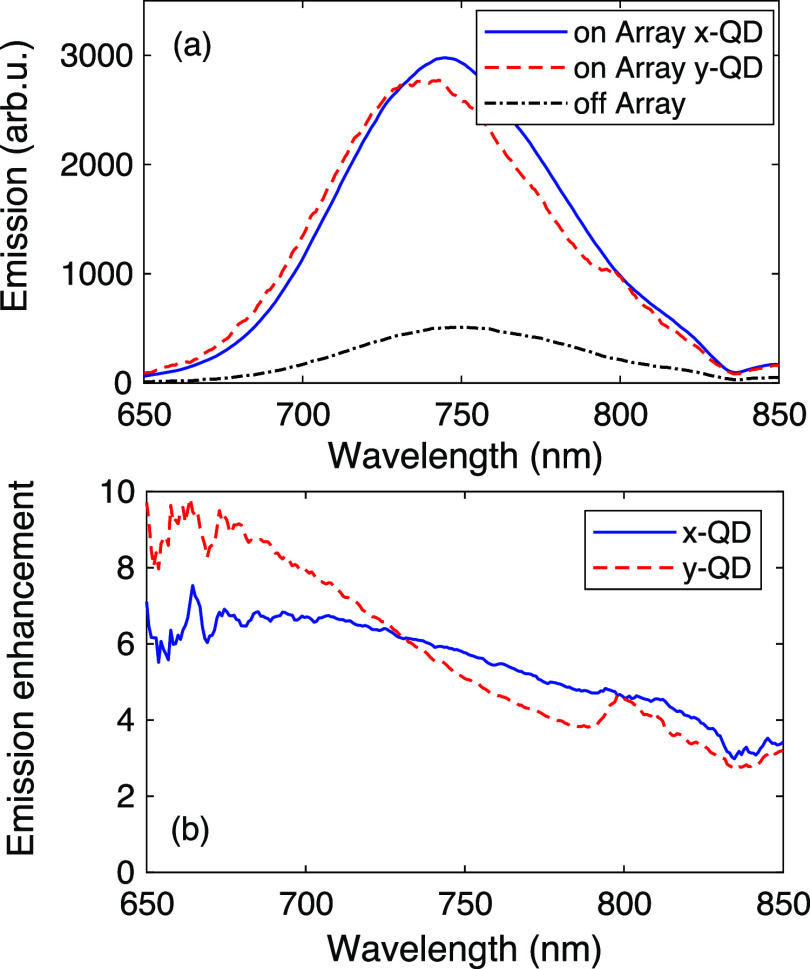
(a) Emission spectra of InP/ZnS QDs on a glass/Si substrate (dash–dotted line) and on the nanoantenna array, detected with polarization analyzer along the *x*-axis (x-QD, solid line) and the *y*-axis (y-QD, dashed line). (b) Emission enhancement factor for x-QD (solid line) and y-QD (dashed line). The excitation source is a continuous-wave laser at 514 nm.

To investigate this further, we measured the decay dynamics of x-QDs and y-QDs under x-EX and y-EX excitation using the 657 nm laser. Accordingly, four distinct cases were considered, as schematically illustrated in Figures 5a–5d. Figure 5a,5b correspond to x-EX, with the QD emission polarized along the *x*-axis (x-QD) and *y*-axis (y-QD), respectively. Under these conditions, the laser weakly excites the B-SC, while excitons in the QDs can excite both the B-SC and the E-SC. On the other hand, Figure 5c,d correspond to y-EX, again with x-QD and y-QD emission, respectively. In the case shown in Figure 5c, the laser primarily excites the E-SC, while the excitons preferentially excite the B-SC. Finally, in Figure 5d, both the laser and the excitons favor excitation of the E-SC.

**Figure 5:**
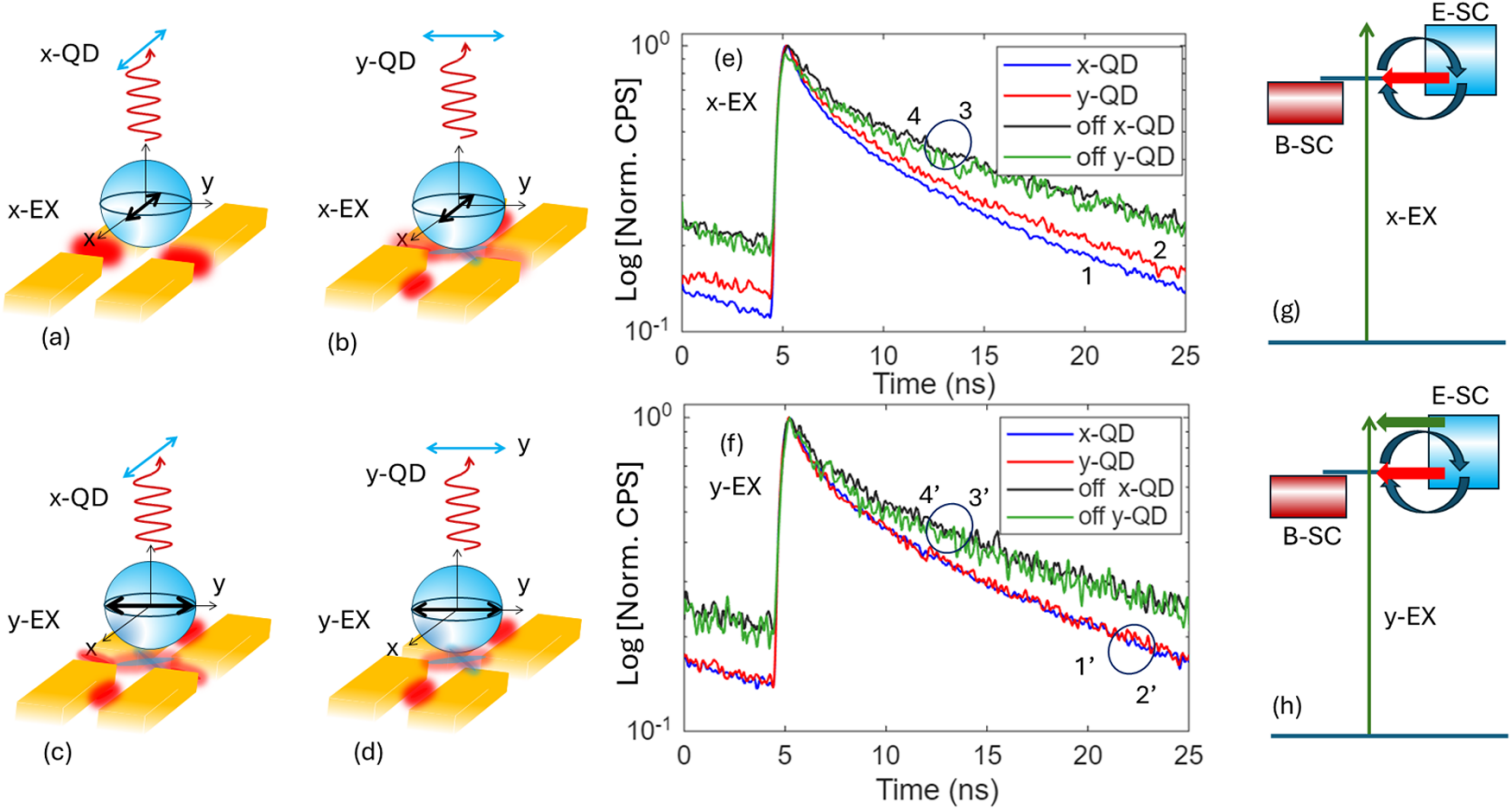
Schematics of sample excitation with a 657 nm laser polarized along the *x*-axis (x-EX) ((a), (b)) and the *y*-axis (y-EX) ((c), (d)). Black double-headed arrows indicate the excitation polarization, and blue arrows denote x-QD and y-QD emission. Panels (e) and (f) show QD decay dynamics under x-EX and y-EX, respectively. In (e), lines 1 and 2 correspond to x-QD and y-QD on the nanoantenna array, while lines 3 and 4 show the corresponding decays on the glass/Si substrate. In (f), lines 1′–4′ correspond to lines 1–4 in (e) but under y-EX excitation. All decay traces were recorded at an emission wavelength of 740 nm. Panels (g) and (h) schematically illustrate hot-electron injection into the QDs under x-EX and y-EX excitation, with red arrows indicating exciton-induced contributions and green arrows indicating optically generated hot electrons.

Figure 5e shows the time-resolved emission decay of QDs at 740 nm under x-EX. One can see here that the decay of x-QD (line 1) is noticeably faster than that for y-QD (line 2). As discussed below, this trend is consistent with the results in Figure 4 and reflects the influence of hot-electron generation and subsequent charging of QDs’ environment. Specifically, the weaker hot-electron effects result in shorter QD lifetimes, whereas stronger hot-electron impact–more prominent for y-QD emission–tends to prolong the decay. In contrast, for QDs located off the array (on the glass/Si substrate), the decay dynamics are polarization-independent (lines 3 and 4). The much longer decay in this case is associated with lack of energy transfer from QDs to F-nanos and absence of Purcell effect.

As shown in Figure 5f, under y-EX (Figure 5c,5d), the decay traces of the x-QD and y-QD emissions become nearly identical (lines 1′ and 2′). This behavior indicates that y-EX efficiently excites plasmonic hot spots and surface lattice resonances, leading to enhanced hot-electron generation. These hot electrons contribute at both stages–during plasmon excitation by the incident field and during subsequent exciton formation–thereby extending the emission lifetime through charge transfer and temporary charging of the QDs’ environment. The similarity between lines 1′ and 2′ highlights the dominant role of direct excitation of E-SC by the laser field and decay of plasmons. The results for the decay are average over four measurements with standard deviation of about 8%.

To quantitatively compare these results, we fitted the decay curves shown in Figure 5e,5f using a biexponential model, (*I* = *A*_*f*_ *e* ^–*a*_*f*_ *t*^ + *B*_*s*_ *e* ^–*b*_*s*_ *t*^) where *A*_*f*_ and *B*_*s*_ are the amplitudes of the fast and slow decay components, respectively, and *a*_*f*_ and *b*_*s*_ are their corresponding decay rates. The fast and slow components are commonly associated with nonradiative and radiative recombination processes, respectively.^19–21^

The fitting results are summarized in Table 1. Compared with line 3, lines 1 and 2 exhibit larger values of *A*_*f*_, indicating a greater contribution from the fast decay channel. In addition, both lines show larger values of *a*_*f*_, corresponding to accelerated nonradiative decay. However, relative to line 1, line 2 exhibits smaller values of both *A*_*f*_ and *a*_*f*_, suggesting a partial suppression of the nonradiative decay process. In contrast, lines 1 and 2 display similar values of *B*_*s*_ and *b*_*s*_, indicating that the slow radiative decay channel is only weakly affected. A similar trend is observed for lines 1′ and 3′.

**Table 1:** Fitting to curve to the date in Figure 5e,5f

	*A*_*f*_	*a*_*f*_ ns^–1^	*B*_*s*_	*b*_*s*_ ns^–1^
Line 3 (4): x-EX x-QD	3.981	0.450	0.76	0.0467
Line 1: x-EX x-QD	10.82	0.604	0.704	0.066
Line 2: x-EX y-QD	6.726	0.516	0.701	0.06
Line 3′ (4′): y-EX x-QD	3.716	0.451	0.786	0.046
Line 1′ (2′): y-EX y-QD	5.913	0.501	0.718	0.059

For the cases shown in Figure 5e,5f, the average excitation intensity was 7.7 mW. To probe the effect of exciton density, Figure 6 compares the QD decay dynamics measured at this intensity and at 0.2 mW. As shown in Figure 6a, when detecting x-QDs, the QD lifetime decreases with increasing excitation intensity, and the difference between x-EX and y-EX excitation becomes more pronounced. At lower exciton densities, this difference is reduced. The shorter lifetime at higher intensity reflects increased nonradiative processes, while the enhanced disparity between x-EX and y-EX excitation indicates polarization-dependent hot-electron generation, with y-EX more efficiently exciting E-SCs and SLRs.

**Figure 6:**
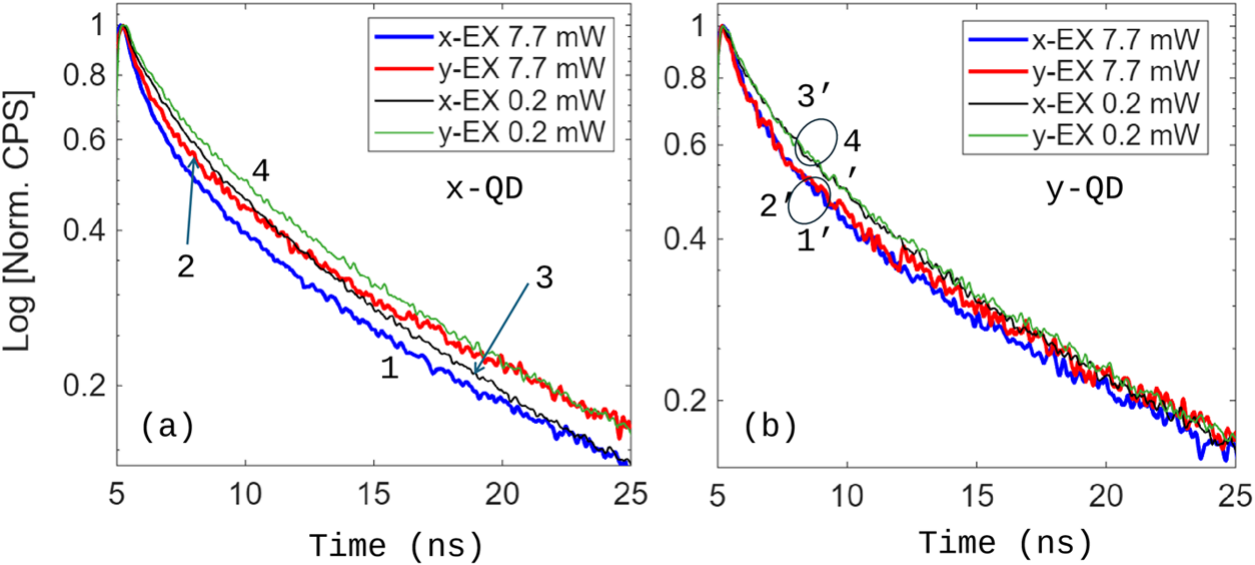
Decay dynamics of x-QDs (a), and y-QDs (b), measured while switching the excitation polarization between x-EX and y-EX. In (a), lines 1 and 2 correspond to x-EX and y-EX excitation, respectively, at an average power of 7.7 mW, while lines 3 and 4 show the same measurements at a reduced power of 0.2 mW. In (b), lines 1′–4′ correspond to lines 1–4 in (a) but for y-QD emission. All decay traces were recorded at an emission wavelength of 740 nm.

In contrast, for y-QDs (Figure 6b), excitons can also couple to and excite the E-SC mode, thereby contributing to hot-electron generation. Consequently, the decay dynamics become nearly independent of the excitation polarization at both 7.7 mW (lines 1′ and 2′) and 0.2 mW (lines 3′ and 4′). A pronounced dependence on excitation intensity, however, remains evident, with higher excitation powers resulting in faster decay rates. The fitting results for the 0.2 mW measurements are provided in the Supporting Information.

## Hot Electrons Lifetime Enhancement and Spectral Shift

### Overall Picture

The impact of x-EX and y-EX excitation is schematically illustrated in Figure 5g,5h. In the case of x-EX, laser excitation leads to a relatively low level of hot-electron generation. However, following exciton formation and subsequent coupling to the y-QD channel (Figure 5b), excitation of the E-SC mode generates additional hot electrons (red arrow in Figure 5g). In the case of y-EX, the E-SC mode is directly excited by the incident laser field. Consequently, hot electrons are generated through direct optical excitation (green arrow in Figure 5h). Similar to the x-EX case, exciton formation is followed by QD-induced excitation of the E-SC mode, resulting in further hot-electron generation (red arrow in Figure 5h).

These processes are strongly influenced by plasmonic hot-spots and lattice-mediated collective fields associated with SLRs, which are known to increase hot-carrier generation and enhance photocurrent or photochemical activity compared to isolated nanoparticles.^13–15^ Such enhancement arises through several mechanisms, including improved light trapping and absorption at resonance, prolonged plasmon lifetimes that increase the energy available for hot electron excitations, and the coherent distribution of excitation across multiple nanoparticles. Previous studies have shown that these effects facilitate collective photoemission and charge transfer into neighboring semiconductors or molecular layers.^1,22,23^

The role of hot electrons and plasmonic fields in the structure investigated in this work is illustrated schematically in Figure 1g. The figure depicts hot-electron generation within the plasmonic nanostructures, their subsequent transfer into the Si interlayer, and their eventual trapping at defect sites located near the QDs. Here, *k*_*th*_ denotes the hot-electron thermalization rate, while *k*_*h*_ represents the rate of hot-electron transfer across the Au/Si interfacial barrier. In addition, *k*_*f*_ in Figure 1g is defined as the rate at which photoexcited electrons escape from the QD core. Figure 1g also schematically illustrates the presence of out-of-core defect sites within the Si layer in close proximity to the QDs.

In addition to these extrinsic defect states, InP/ZnS QDs are known to exhibit a relatively high probability of defect formation at the core/shell interface.^24–26^ These defects can introduce electronic states within the bandgap, giving rise to red-shifted emission tails and broader photoluminescence spectra.^24^

Occupation of such out-of-core defect sites by hot electrons alters the emission dynamics in a manner distinct from the charging effects associated with core-localized charges. The latter primarily occurs during optical excitation of the QDs, when photoexcited electrons migrate from the QD cores to out-of-core defect sites, leaving holes confined within the cores. This process is characterized by *k*_*f*_ in Figure 1g. As a result, the emission lifetime is reduced through enhanced Auger recombination, leading to the formation of dark QDs.^27,28^

Occupation of out-of-core defect sites, either through the hot-electron transfer mechanism illustrated in Figure 1g or through charge migration from other QDs that have become dark, results in the formation of an external Coulomb field. This field perturbs the potential landscape experienced by the photoexcited electron–hole pair within the QD core.^29–32^ Consequently, the photoexcited electron experiences a repulsive potential, whereas the hole is weakly attracted toward the QD surface. This partial spatial separation of the charge carriers reduces the electron–hole wave function overlap, leading to a decrease in both the oscillator strength. As a result, the radiative lifetime increases modestly to some extent.

Figure 7 shows calculations for a simplified electrostatic model in which a positive and a negative charge are separated by 10 nm, depicting the configuration depicted in Figure 1g.^32^ The figure includes the approximate dimensions of the InP/ZnS core, ZnS shell, and ligand layer, as well as a schematic of a photoexcited electron–hole pair confined within the QD core. The calculated potential distribution illustrates that the electrostatic field extends over length scales comparable to the dimensions of the QD, indicating that trapped charges can perturb the excitonic states. Here the influence of the surrounding Au nanostructures and Si layer on the electrostatic field is neglected in this simplified treatment.

**Figure 7:**
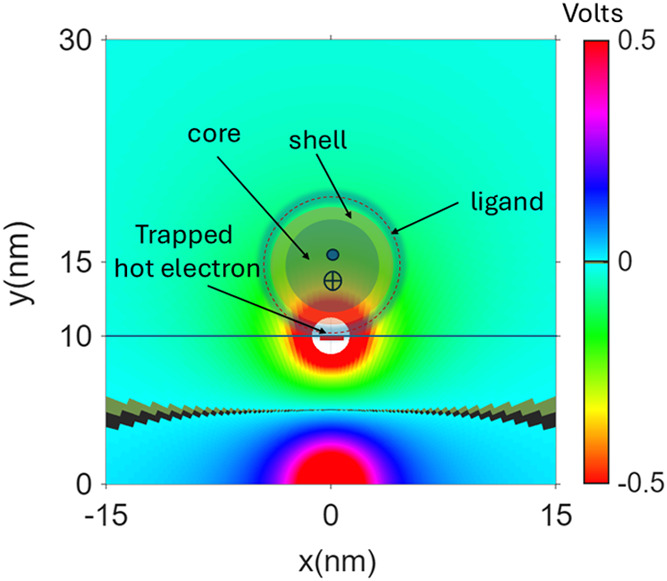
Electrostatic potential associated with an positive and negative charges separated from each other by 10 nm. A InP/ZnS QD with schematics for core, shell, and ligand are placed on the top of the 10 nm scale, depicting the interface of Si layer in the structure studied in this paper. The vertical color-coded bar show the scale of potentials in Volts. The figure also highlights the postion of an electron/pair in the presence of the field.

### Plasmonic Model for Hot Electron-induced Dynamics

The electrostatic fields generated by out-of-core charges, namely hot electrons trapped at the QD/Si interface (Figure 1g), can significantly alter the decay dynamics of the QDs. This effect arises because such fields reduce the probability of QD core charging by photoexcited carriers. Specifically, the external Coulomb field suppresses the migration of photoexcited electrons from the QD core to out-of-core defect states (*k*_*f*_), including defects located in the shell and even in the surrounding substrate.^29–32^ As a result, Auger recombination is mitigated, leading to an increase in the QD emission lifetime.^30,31^

To further examine this mechanism, we adopt a phenomenological model, schematically illustrated in Figure 1g. The model consists of a three-level system in which |*g*⟩ and |*e*⟩ represent the Fermi-sea electron state and the hot-electron state, respectively. The third state, |*d*⟩, corresponds to hot electrons trapped in out-of-core defect states. We consider the case in which the optical field directly excites the plasmonic nanostructure, analogous to the excitation scenario shown in Figure 5h. The hot-electron generation rate is therefore given by *k*_*exc*_ = *ησ*_*abs*_ *I*/(ℏω_*l*_), where σ_abs_ is the absorption cross section of the plasmonic structure, *I* is the incident optical intensity, ℏω_*l*_ is the photon energy of the excitation laser, and η denotes the efficiency of plasmon-to-hot-electron conversion. The hot-electron state |*e*⟩ decays through thermalization and transfer processes with rates *k*_*th*_ and *k*_*h*_, respectively. The defect state |*d*⟩ is populated through the transfer rate *k*_*h*_ and depopulated through a comparatively slow neutralization process.

The Coulomb field associated with a trapped hot electron modifies the local potential landscape and suppresses subsequent electron-transfer processes (Figure 7). In particular, it reduces both the hot-electron transfer rate into the Si layer, *k*_*h*_, and the rate of photoexcited-electron migration from the QD core, *k*_*f*_. Introducing Φ_*d*_ as the occupation probability of the trapped-electron state |*d*⟩, these transfer rates can be expressed in terms of renormalized rates according to^29,30^

1 $$k_{f}^{\prime }=k_{f}\left(1-\Phi_{d}\right)k_{h}^{\prime }=k_{h}\left(1-\Phi_{d}\right)$$

Here *k*_*f*_^′^ and *k*_*h*_^′^ are the rates influenced by Φ_*d*_. Here we considered effectively only one QD is influenced by a single trapped hot electron.

On the other hand, Φ_*d*_ depends on the efficiency of hot-electron transfer into the Si layer. To obtain Φ_*d*_, we note that the decay rate of state |*d*⟩, characterized by a neutralization rate, is ignored. Therefore, the population of |*d*⟩ can accumulate over time, and its dynamics are approximately described by the following rate equation:

2 $$\frac{d\Phi_{d}}{\mathrm{dt}}=\left(1-\Phi_{d}\right)k_{h}\Phi_{e}$$

In this equation Φ_*e*_ represent the probability of hot-electron in state |*e>*. Under steady state illumination, it can be obtained from the following:

3 $$\Phi_{e}=\left(1-\Phi_{d}\right)/\left(\frac{k_{\mathrm{th}}+k_{h}^{\prime }}{k_{\mathrm{exc}}}+1\right)$$

For the case of Au nanoantennas, hot-electron injection (*k*_*h*_) has been reported to occur on time scales of approximately 50 fs.^33–35^ On the other hand, electron–electron and electron–phonon scattering, which constitute the dominant thermalization pathways for hot electrons, are reported to occur on time scales of approximately 100 fs and 1 ps, respectively.^33,36^ Therefore, for the simulations, we consider *k*_*h*_^–1^ to be approximately 50 fs and *k*_*th*_^–1^ to be approximately 500 fs. Additionally, we assume η∼0.01.^37,38^

To proceed with the analysis of the QD emission dynamics, it is necessary to consider the influence of plasmon resonances on both radiative and nonradiative decay processes. While the electromagnetic environment of the F-nano arrays is inherently complex, a simplified model consisting of a single Au nanorod (NR) and a nearby QD (Figure 8a) can provide valuable physical insight (see the Supporting Information for details). The primary purpose of this model is to estimate the relative strengths of plasmonic field enhancement and energy transfer from the QD to the metallic nanoantenna. Although the model neglects SLR formation and does not accurately reproduce the geometric scales of the actual arrays studied here, its simplicity permits analytical evaluation of the Purcell effect and the QD-to-metal energy-transfer rate, i.e., Forster resonance energy transfer (FRET). These estimates serve as a useful benchmark for interpreting the experimentally observed decay dynamics.

**Figure 8:**
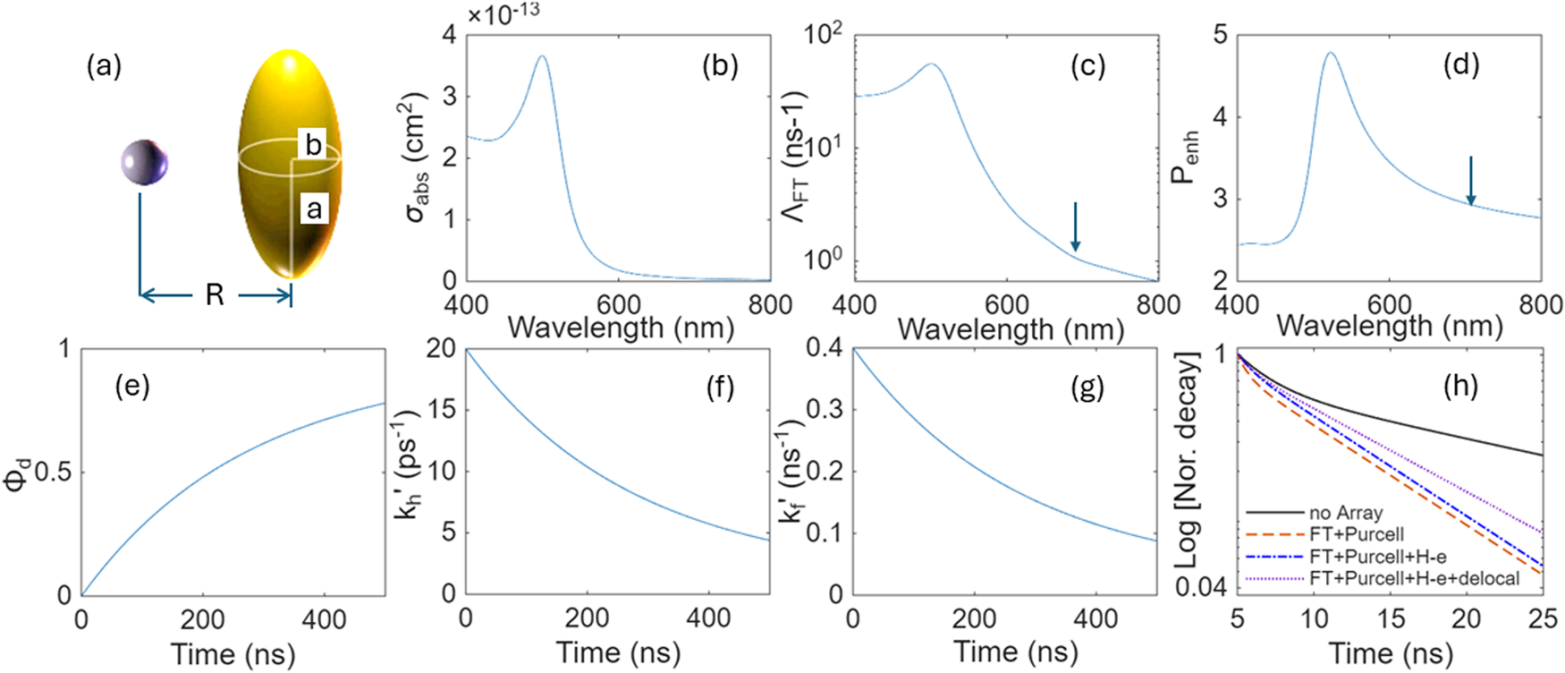
(a) Schematic of the QD–NR system. (b)–(d) Calculated absorption cross section, QD-to-NR FRET rate, and plasmonic field-enhancement factor, respectively. (e)–(g) Time-dependent variations of Φ_*d*_, *k*_*h*_^′^, and *k*_*f*_^′^. (h) Calculated QD decay dynamics. Here solid line corresponds to QDs without NRs (lines 3 and 4 in Figure 5a); dashed line includes FRET and *P*_*enh*_; dash-dotted line includes hot-electron transfer (*k*_*f*_^′^); and dotted line includes the suppression of the radiative decay rate by the hot-electron-induced electrostatic field.

In the following, we approximate the NR as a spheroid with *a* = 10 nm, *b* = 5 nm, and *R* = 15 nm (Figure 8a). We assume that the NR is embedded in Si, while the QD is located in air. The results shown in Figure 8b present the absorption cross section of the NR as a function of wavelength. Since the polarization of the incident field is assumed to be aligned along the *b* axis, the observed resonance corresponds to the transverse mode of the NR. Figures 8c and 8d show the FRET rate from the QD to the NR (Λ_*FT*_) and the field-enhancement factor at the position of the QD (*P*_*enh*_), respectively. Both quantities exhibit maxima near 500 nm.

To estimate the relevant operating wavelength for the purposes of this paper, we note that the experimental observations presented in Figures 3 and 4 indicate an enhancement of the QD emission. Such behavior requires relatively weak FRET accompanied by a moderate plasmonic field enhancement. The results in Figure 8c,8d show that, at shorter wavelengths, *P*_*enh*_ increases only modestly, whereas Λ_*FT*_ increases dramatically (note that the Λ_*FT*_ axis in Figure 8c has logarithmic scale). Under these conditions, energy transfer from the QD to the NR is expected to dominate, leading to quenching of the QD emission. Furthermore, the experimental results shown in Figure 2f suggest a value of *P*_*enh*_ ≈ 3.

Based on these considerations, we evaluate Λ_*FT*_ and *P*_*enh*_ at 700 nm (arrows in Figures 8c and 8d), obtaining Λ_*FT*_ ≈ 1 ns^–1^ and *P*_*enh*_ ≈ 3. From the fitting results for QDs off the array (Table 1, Line 3 (4)), we estimate *k*_*f*_ ≈ 0.4 ns^–1^. Combined with the results of Figure 8g, we consider *k*_*f*_^′^ ≈ 0.1 ns^–1^. The pronounced reduction in *k*_*f*_^′^ demonstrates that the hot-electron-induced Coulomb field strongly suppresses electron escape from the QD core.

Solid line in Figure 8h shows the fit obtained for lines 3 and 4 in Figure 5a. Dashed line, however, corresponds to the case in which the effects of both FRET and Purcell enhancement are included. To account for these processes, the nonradiative decay rate (*a*_*f*_ in Table 1) was increased by 1 ns^–1^, while the radiative decay rate (*b*_*s*_ in Table 1) was increased by a factor of 3. Such behavior is commonly observed in QD–nanoantenna systems, where plasmonic structures simultaneously enhance radiative emission and introduce additional nonradiative decay channels.^19,31,39,40^ The fit represented by dashed line does not include the influence of hot electrons but does include the effect of *k*_*f*_^′^, corresponding to the transfer of photoexcited electrons from the QD core to defect sites.

In the presence of hot electrons, we reduce this rate from 0.4 to 0.1 ns^–1^, according to the results shown in Figure 8g. This modification generates dashed-dotted in Figure 8h. The comparison between dashed and dashed-dotted line, however, does not reproduce the trend observed experimentally in Figure 5e (lines 1 and 2). This discrepancy can be attributed to the fact that the dashed-dotted line does not account for the influence of the trapped-charge electric field on the radiative lifetime of the exciton. As discussed previously, the electrostatic field generated by trapped hot electrons is expected to induce a partial spatial separation between the electron and hole wave functions. This reduces their overlap and consequently decreases the radiative decay rate. To incorporate this effect, we further reduce the radiative decay rate via delocalization of electron/hole pair by 0.5 ns^–1^. The resulting fit is shown as the dotted line in Figure 8h.

A comparison of solid and dotted line in Figure 8h now reproduces the qualitative behavior observed experimentally between lines 1 and 2 in Figure 5e. These results suggest that the experimentally observed increase in the emission lifetime cannot be explained solely by suppression of electron transfer to defect states. Rather, it arises from the combined effects of reduced Auger-related losses and a modest decrease in the radiative decay rate caused by the electrostatic field of trapped hot electrons.

Considering these results, the blueshift observed in Figure 4 can be understood as a consequence of hot-electron-induced suppression of carrier trapping. In the absence of hot electrons, photoexcited electrons can migrate from the QD core to low-energy defect states located at the core/shell interface or in nearby out-of-core defects. Radiative recombination involving these states contributes predominantly to the red side of the emission spectrum.^24,26^ When hot electrons occupy these defect states, the associated Coulomb field suppresses further electron transfer into them, effectively reducing the rate k_*f*_. This allow the photoexcited electrons recombine with holes at higher energies.

## Conclusions

We have shown that surface lattice resonances (SLRs) and plasmonic hotspots in periodic arrays of flat Au nanoantennas provide an effective mechanism for controlling the emission dynamics of InP/ZnS quantum dots through hot-electron-mediated interactions. Hybridization between localized plasmon modes and Rayleigh anomalies generates long-lived collective resonances with enhanced optical absorption, facilitating efficient hot-electron generation and transfer. Through polarization-selective excitation, distinct plasmonic supercells can be activated, with y-polarized excitation producing stronger SLR-mediated hot-electron injection across the Au/Si interface and enhanced photocharging of the QD environment. The resulting electrostatic fields modify carrier trapping and recombination pathways within the QDs, leading to measurable changes in both emission lifetime and spectral position. These results underscore the central role of lattice-mediated plasmonic fields in tailoring exciton–plasmon coupling and demonstrate a promising strategy for dynamically controlling light emission in hybrid plasmonic–semiconductor nanostructures.

## Supplementary Material



## Notes

1. Fitting to decay with 0.2 mW average intensity; and 2. Modeling of FRET and Purcell effect (PDF)

## §

Salience Laboratories, United Kingdom

## ∥

Wiley, United Kingdom
