# Supplementary material for: Hot Electrons Control of Quantum Dot Emission Using Plasmonic Supercells
Source: J Phys Chem C Nanomater Interfaces. 2026 Jul 13;130(29):10477–86. doi: 10.1021/acs.jpcc.6c03809 (PMC13403302; doi:10.1021/acs.jpcc.6c03809)
Supplement: Supplementary file 1 [file jp6c03809_si_001.pdf]

Supporting Information

Hot Electrons Control of Quantum Dot Emission Using Plasmonic Supercells

Seyed M. Sadeghi,[1] Rithvik Gutha,[1] Christina Sharp,[1] Ryan W. Goul,[2] and Judy Wu[2]

[1]Department of Physics and Astronomy, University of Alabama in Huntsville, Huntsville, Alabama, 35899, USA

[2]Department of Physics and Astronomy, The University of Kansas, Lawrence, Kansas, 66045, USA

1-Fitting to decay with 0.2 mW average intensity:

The results of bi-exponential fitting to the data in Fig. 6 for average excitation intensity of 0.2 mW at 657 nm wavelength.

	A_f	a_f (ns^{-1})	B_s	b_s (ns^{-1})
Line 4: y-EX x-QD	0.592	0.049	2.365	0.299
Line 3: x-EX x-QD	0.492	0.048	2.367	0.276
Line 3' (4') y-EX y-QD	0.535	0.046	2.100	0.263

2-Modeling of FRET and Purcell Effect:

Fig. 8a shows the plasmonic system considered for calculation of FRET and Purcell effect. In this system NR is treated classically with an induced dipole moment described by an energy-dependent scalar polarizability. This polarizability can be controlled by shape, size and the dielectric contrast between the metal nanostructure and the nonconductive environment. We model the NR as an elongated spheroid where the corresponding aspect ratio is $q = a/b$. The polarizability can be written as:[1]

$$\beta(\omega) = \frac{[\varepsilon_{NR}(\omega) - \varepsilon_b]}{[3\varepsilon_b + 3\kappa(\varepsilon_{NR}(\omega) - \varepsilon_b)]} \quad (1)$$

In Eqn. (1), κ is called the depolarization factor of the NR,$\varepsilon_{NR}(\omega)$ and $\varepsilon_b(\omega)$ are the dielectric constants of the NR and the local background around it, respectively. The depolarization factor is considered the same for both configurations. This factor is defined as [2]:

$$\kappa = \frac{1-e^2}{e^2}\left[\frac{1}{2e}\ln\left(\frac{1+e}{1-e}\right)-1\right] \quad (2)$$

where $e = \sqrt{1-1/q^2}$. As shown in Ref. 2, based on this the FRET rate from QD to the NR can be given as:

$$\Lambda_{FT} = \frac{ab^2 S_a^2 \mu_{21}^2 \beta(\omega)}{\hbar \varepsilon_{eff}^2 R^6} \quad (3)$$

Here for the polarization of the incident light in this paper S_a=2. The enhancement factor, defined as the ratio of field in the presence of NR to that to its absence is given as:

$$P_{enh} = |1 + \frac{ab^2 S_a \beta(\omega)}{R^3}|^2 \quad . \quad (4)$$

1) Link, Stephan, Mona B. Mohamed, and M. A. El-Sayed. "Simulation of the optical absorption spectra of gold nanorods as a function of their aspect ratio and the effect of the medium dielectric constant." *The Journal of Physical Chemistry B* 103, no. 16 (1999): 3073-3077.
2) Hatef, Ali, S. M. Sadeghi, and Mahi R. Singh. "Coherent molecular resonances in quantum dot–metallic nanoparticle systems: coherent self-renormalization and structural effects." *Nanotechnology* 23, no. 20 (2012): 205203.
